# The impact of metabolism on the adaptation of organisms to environmental change

**DOI:** 10.3389/fcell.2023.1197226

**Published:** 2023-06-12

**Authors:** Douglas L. Rothman, Peter B. Moore, Robert G. Shulman

**Affiliations:** ^1^ Departments of Radiology, Yale University, New Haven, CT, United States; ^2^ Biomedical Engineering, Yale University, New Haven, CT, United States; ^3^ Yale Magnetic Resonance Research Center, Yale University School of Medicine, New Haven, CT, United States; ^4^ Department of Molecular Biology and Biophysics, Yale University, New Haven, CT, United States; ^5^ Department of Chemistry, Yale University, New Haven, CT, United States

**Keywords:** adaptation, metabolism, gene expression, metabolic plasticity, Crabtree effect, lac operon, glycogen shunt

## Abstract

Since Jacob and Monod’s discovery of the lac operon ∼1960, the explanations offered for most metabolic adaptations have been genetic. The focus has been on the adaptive changes in gene expression that occur, which are often referred to as “metabolic reprogramming.” The contributions metabolism makes to adaptation have been largely ignored. Here we point out that metabolic adaptations, including the associated changes in gene expression, are highly dependent on the metabolic state of an organism prior to the environmental change to which it is adapting, and on the plasticity of that state. In support of this hypothesis, we examine the paradigmatic example of a genetically driven adaptation, the adaptation of *E. coli* to growth on lactose, and the paradigmatic example of a metabolic driven adaptation, the Crabtree effect in yeast. Using a framework based on metabolic control analysis, we have reevaluated what is known about both adaptations, and conclude that knowledge of the metabolic properties of these organisms prior to environmental change is critical for understanding not only how they survive long enough to adapt, but also how the ensuing changes in gene expression occur, and their phenotypes post-adaptation. It would be useful if future explanations for metabolic adaptations acknowledged the contributions made to them by metabolism, and described the complex interplay between metabolic systems and genetic systems that make these adaptations possible.

## Introduction

The mechanisms that enable organisms to adapt metabolically to sudden alterations in their environment have been studied for over a century. Much of this work has been directed at understanding the changes in levels of gene expression that often occur when organisms are challenged this way. Consequently, and not surprisingly, most of the explanations offered for these adaptations have assigned a determinative role to genetic regulation. The paradigm for these models is a product of Monod’s and Jacob’s ground-breaking studies of lactose metabolism in *Escherichia coli* which led to the discovery of the lactose operon and the lactose repressor protein (Jacob and Monod, 1961). When the lac system was first elucidated, the flow of information that makes it possible for *E. coli* that has been growing on glucose to adapt to using lactose appeared to be very simple. A change in the external environment, in this case, exhaustion of the supply of glucose, results in a modification of the state of a protein that regulates the expression of relevant genes, i.e., the lac repressor and the lac operon, respectively. The resulting change in rates at which those genes are expressed enables the cells to resume growth in their new environment. In this paper, we argue that the role played by metabolism in metabolic adaptations has been under-appreciated. We will review results which demonstrate that metabolism often plays a major role in adaptation, commonly interacting strongly with gene expression systems. We then show not only that a better understanding of metabolic adaption emerges when these interactions are taken into account, but also that their inclusion can shed light on the results of earlier experiments going back to Monod’s original work on sugar metabolism in bacteria.

Before we review evidence for the importance of the role of metabolism in adaptation, we define the terms we will use to describe models of how organisms adapt to sudden environmental changes. For convenience, we will describe models for metabolic adaptation that ascribe it predominantly, if not exclusively, to changes in gene expression as “genetically driven.” Elsewhere in the literature models of this sort are often referred to as “top-down” (Lewontin et al., 1984; Craver, 2007). Further, we will refer to models for adaptive processes that assert that outcomes are determined primarily by the properties of the metabolic system that was in place in an organism before its environment changed as “metabolically driven.”

The degree to which genetically driven models for adaptation have come to permeate the biological literature hardly needs to be described here. For example, the premise that adaptation is genetically driven has been fundamental to much of the research that has been done on the human genome (Collins and McKusick, 2001). Our assertion that metabolism, in effect, “co-authors” adaptation is consistent with a long-running storyline in the adaptation field that has commonly been underemphasized, if not ignored outright. Evidence has long existed that organisms can adapt to major environmental changes for extended periods of time, independent of any changes in gene expression (Rothman et al., 2021). For example, within fractions of a second, exercise can trigger many fold increases in the flux through the metabolic pathways that supply muscle with energy, and these increases can be sustained for hours without significant changes in levels of gene expression of (Hargreaves, 2015). Even more difficult to reconcile with a pure genetically driven view of adaptation are the many discoveries in recent decades of molecular mechanisms that enable metabolic enzymes and intermediates to control gene expression (e.g., Van der Knapp and Verrijzer, 2016; Kochanowski et al., 2017; Li et al., 2018; Wang and Lei. 2018).

Even though there is clear evidence that there are aspects of metabolic adaptation that genetically driven models cannot explain, it has had little impact. For all intents and purposes, in fields ranging from molecular to evolutionary biology, alterations in levels of gene expression are presented as the sole drivers of metabolic adaptation. In medical science, rapid metabolic adaptations and maladaptations, e.g., the elevation in blood glucose seen in diabetics, are well known and well studied. Nevertheless, the continued emphasis of the NIH and medical science in general on finding genetic explanations for metabolic diseases (Collins and McKjusick, 2001; Tragante et al., 2014; DeForest and Majithia, 2022) has led to neglect of the possibility that metabolism might play a role in them too.

We agree with previous critiques that the almost exclusive focus in some fields on genetic driven adaptation mechanisms has been a barrier to progress (Lewontin et al., 1984; Fox-Keller, 1991; McKnight, 2010). However, our approach is epistemologically pragmatic (Peirce, 1958; Craver, 2007). It is our thesis that investigators interested in understanding metabolic adaptations need to take account not only the contributions that changes in gene expression make to them, but also the enabling role played by metabolism, which includes its impact on the regulation of gene expression. To support this thesis, we will discuss two well-studied metabolic adaptations: the Crabtree effect (De Deken, 1966), which is seen when yeast that have been growing oxidatively on poor substrates are suddenly presented with glucose, and the diauxic growth that occurs when *E. coli* that has been growing on both glucose and lactose runs out of glucose (Monod, 1941; Jacob and Monod, 1961). We have chosen them because the Crabtree effect was long believed to be metabolically driven, and the capacity of *E. coli* that has been growing on glucose to adapt to growth on lactose is the paradigm for genetically driven adaptation. Here we will show that neither can be fully understood without taking account of the strong interplay between the metabolic and genetic systems of these organisms that occurs during the period when they are transitioning from their initial steady states to their final, fully adapted steady states, which we will refer to as the transition phase.

### The transition phase

Neither of the adaptations we are about to discuss is instantaneous. It takes about 2 h for yeast experiencing the Crabtree effect to become fully adapted to growth on glucose, and it takes about 45 min for *E. coli* to switch from log phase growth on glucose to log phase growth on lactose, a phenomenon oftened referred to as a diauxic shift (Monod, 1941). A similar lag during the diauxic shift to galactose metabolism takes place in yeast (Siegal, 2015). These lags are proof positive that both adaptations have transition phases, as do many others.

During the first stage of any transition phase, the existing metabolic apparatus of the cell is almost always suboptimal for growth under the new conditions because it was optimized for growth under the conditions that prevailed before the environment changed, However, and quite obviously, in order for the cell to adapt to the new conditions, its existing metabolic systems must be able to keep it alive long enough for adaptation to occur. We will refer to the capacity of metabolic systems to adapt adequately under a range of external conditions as metabolic plasticity. Under most conditions, changes in the properties of existing enzymes caused by mechanisms such as phosphorylations, dephosphorylations, and allosteric mechanisms are needed to give cells the metabolic plasticity they require, but, importantly, adjustments like these can occur in seconds to a few minutes, long before changes in gene expression can take place.

The second stage of the transition phase is characterized by the changes in gene expression that will be needed to provide the cell with the optimal metabolic system for growth under the new conditions. Tens of minutes or more are required for this to happen. As we will see, these changes are often triggered by alterations in the intracellular milieu caused by the activities of the suite of enzymes that was present in the cells during the first stage of the transition.

**FIGURE 1:**
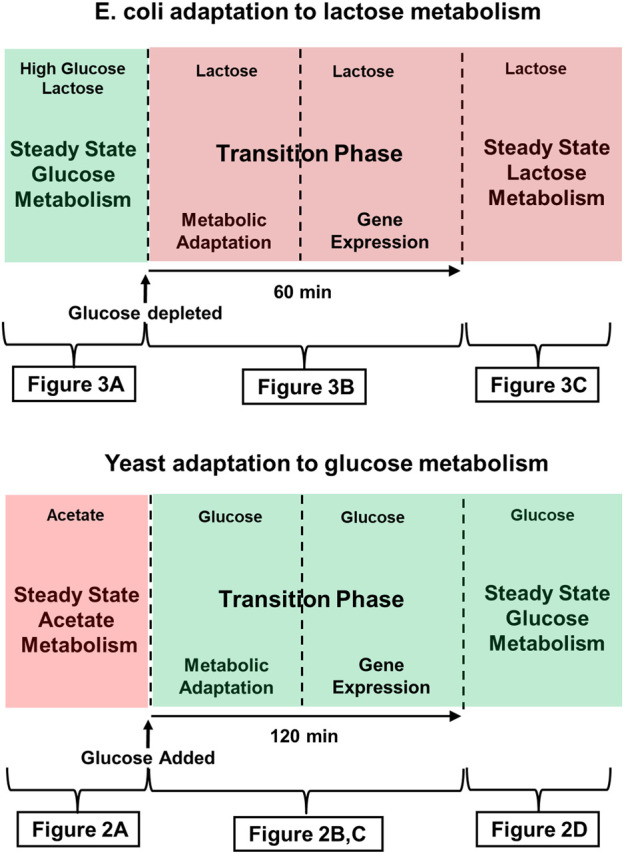
Time course of adaptation to glucose metabolism in Crabtree yeast (e.g., *S. Cerevisiae*, and lactose metabolism in *E. coli*. Green shading indicates that the medium contains glucose, the preferred substrate. Red shading indicates that a less desirable substrate is being metabolized. The figures indicated below each panel provide more detailed descriptions of the metabolic and genetic events characteristic of those stages of adaptation. (Top) The Crabtree effect: Initially, yeast are either in stasis or growing slowly, as they metabolize a poor substrate such as acetate or ethanol oxidatively. Addition of glucose to the medium triggers the Crabtree effect, which ends when the yeast begin growing in log phase, supporting themselves largely by fermenting glucose. (Bottom) *E. coli* adaptation to lactose metabolism: This diagram describes the time course of the diauxic shift seen when *E. coli* that has been growing on glucose adapts to growth on lactose after the supply glucose runs out. During an approximately 60 min transition phase, growth ceases, and by the time growth resumes, cells have begun expressing the genes of the lac operon, the products of which are required for lactose metabolism.

It follows that it is appropriate to describe both of the metabolic adaptations we will discuss here, as well as many others, using the scheme outlined in Figure 1. There is invariably an initial steady state and a final steady state, and those two states are separated by a transition phase that has a first, metabolic stage, and a second, gene expression stage.

### Transcription phase description of the crabtree effect

#### The early history of the Crabtree effect as a metabolically driven adaptation, and results that have challenged this paradigm

In 1928, Herbert Grace Crabtree (1928) discovered that when glucose is supplied to baker’s yeast, *S. cerevisae*, that has been growing on non-glucose substrates in the presence of oxygen, they stop metabolizing those substrates, and immediately begin using glycolysis to meet their needs for both energy and metabolic precursors. Under these conditions, even though there is oxygen present, much of the glucose they consume is converted into ethanol, i.e., fermented, rather than being oxidizing all the way to CO_2_ and H_2_O. One of the most striking features of this response is that the lion’s share of the increase in the flux through the glycolytic pathway that occurs as the Crabtree effect unfolds happens within minutes, well before any changes in gene expression have taken place, or, indeed, could have taken place (den Hollander et al., 1986a; den Hollander et al., 1986b).

**FIGURE 2:**
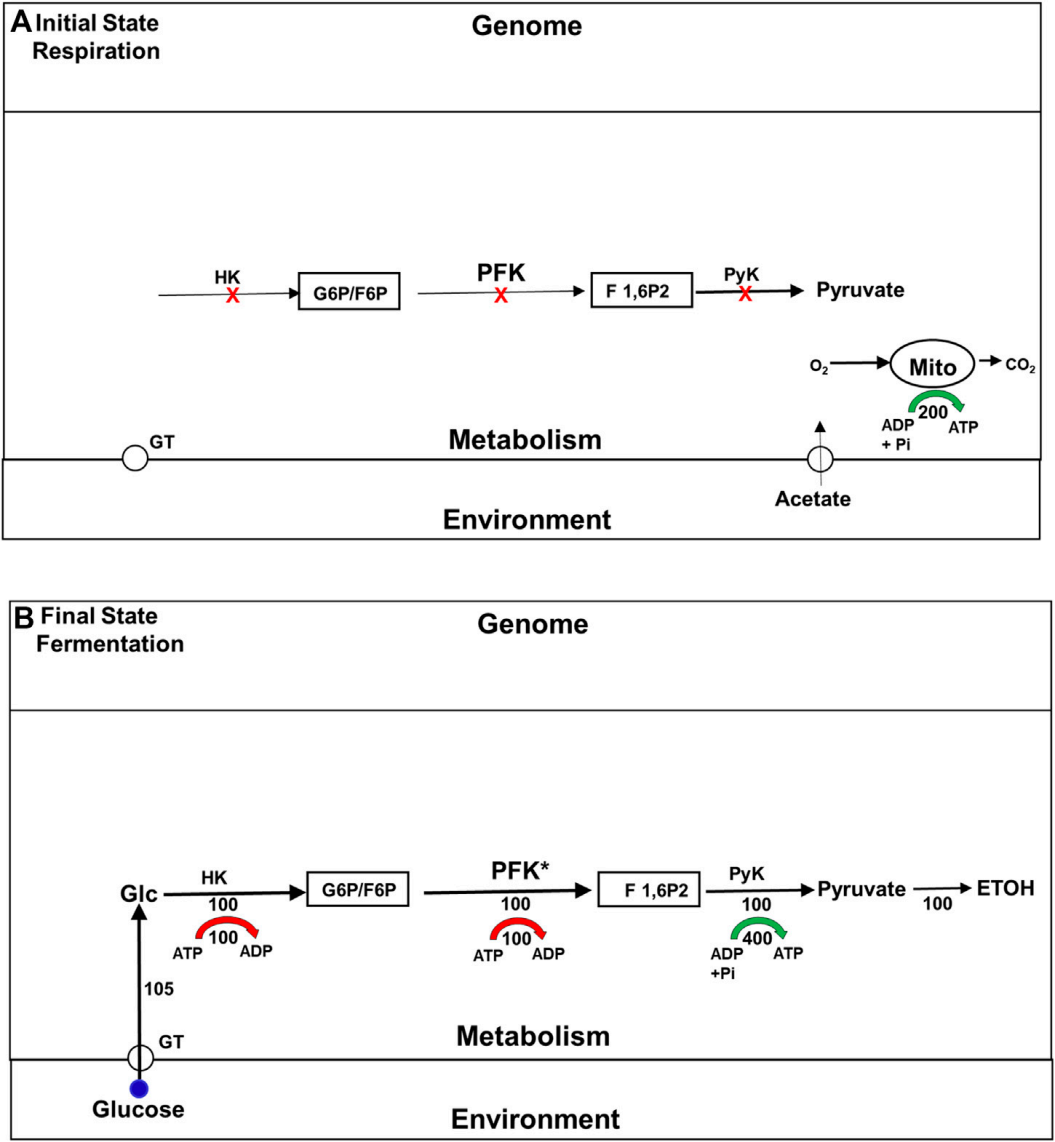
The traditional model for the upregulation of glycolysis and ETOH production during the Crabtree effect. Fluxes are indicated by numbers beneath the corresponding flux arrows. All fluxes are normalized to the rate (100) of phosphofructokinase in the final state of fermentation. Red arrows indicate the ATP consumption fluxes by the HK and PFK reactions, and green arrow indicates ATP production by the distal portion of the glycolytic pathway. **(A)** Initial State (Respiration) Prior to exposure to glucose the cells support themselves by oxidizing substates like acetate to carbon dioxide and water via the tricarboxylic acid cycle (TCA). The glycolytic enzymes are all present at high levels, but inactive because of the lack of glucose, and because phosphofructokinase (PFK), believed to be the rate limiting step, is allosterically inhibited. **(B)** Final State (Glycolysis/Fermentation) Addition of glucose to the medium leads to an elevation of the intracellular glucose concentration, which via hexokinase (HK) leads to an elevation in glucose 6-phosphate (G6P) and fructose 6-phosphate (F6P). The increase in F6P activates PFK both as a substrate and allosterically, leading to production of fructose 1,6 bisphopshate (F 1,6P2). PFK is the rate limiting step in the glycolytic pathway, and therefore determines the rate of the glycolytic flux at steady state (normalized to 100). Most of the glycolytic flux is used to synthesize ethanol (ETOH), i.e., fermented. Abbreviations: ATP, adenosine triphosphate; ADP, adenosine diphosphate; F1,6P2, fructose 1,6-bisphosphate; G6P: glucose 6-phosphate; F 1,6P2, fructose 1,6-bisphosphate; HK: hexokinase; PFK, phosphofructokinase; PyK, pyruvate kinase.

It has long been known that the enzymes involved in the glycolytic pathway are expressed constitutively in yeast. Not surprisingly, therefore, the explanation originally offered for the Crabtree effect was that it is a metabolically driven adaptation that depends almost entirely on the allosteric activation of a single glycolytic enzyme, phosphofructokinase (PFK), the activity of which was believed to be rate limiting for that pathway (Bosca and Corredor, 1984). Figure 2 summarizes this model. [We note in passing that in most educational materials, PFK activity is still identified as rate limiting for glycolysis in yeast and other organisms, even though this is rarely true (Bosca and Corredor, 1984; Rothman et al., 2021)]. The model for the Crabtree effect that is summarize in Figure 2 was called into question in the 1980s and 1990s, by studies that examined the impact mutations and changes in gene expression of glycolytic enzymes have on the rate of ETOH formation. Surprisingly, given its assignment as a rate limiting step, changes in PFK activity had little effect on glycolytic flux (Davies and Brindle, 1992; Boles et al., 1996). Even more surprising, it was found that mutations that block two non-glycolytic pathways, the trehalose and glycogen synthesis pathways, also block the increase in glycolytic rate characteristic of the Crabtree effect (Navon et al., 1979; Thevelein and Hohmann, 1995). Adding to the confusion was the finding that after yeast have been exposed to glucose for about 2 h, the flux through glycolytic pathway remains just as elevated as it becomes minutes after glucose first becomes available, even though the trehalose and glycogen synthesis pathways have been almost completely suppressed. Clearly, under some circumstances these branch pathways are essential for high glycolytic flux, while under others, it is not. Why is that?

Once techniques became available that made it possible to examine changes in rates of gene expression, and to estimate the concentrations of enzyme isoforms in yeast, it became obvious that the Crabtree effect has a genetic component too. About 40 min after glucose addition, major changes in rates of gene expression occur that result in the wholesale replacement of the isoforms of the glycolytic enzyme that were present before glucose became available with different isoforms (Derisi et al., 1997; Van den Brink et al., 2008). Furthermore, even though the kinetic properties of many of these new isoforms are quite different from those of the isoforms they replace, the rate of glucose consumption hardly changes at all, and the fraction of the flux converted into ethanol only increases by approximately 40% (Den Hollander and Shulman, 2005; Rothman et al., 2021). These observations raised a new question. What purpose is served by these isoform changes, as well as the other major changes in gene expression that occur?

#### The importance of metabolic control analysis and related quantitative metabolic models in understanding the regulation of metabolic adaptation

**FIGURE 3:**
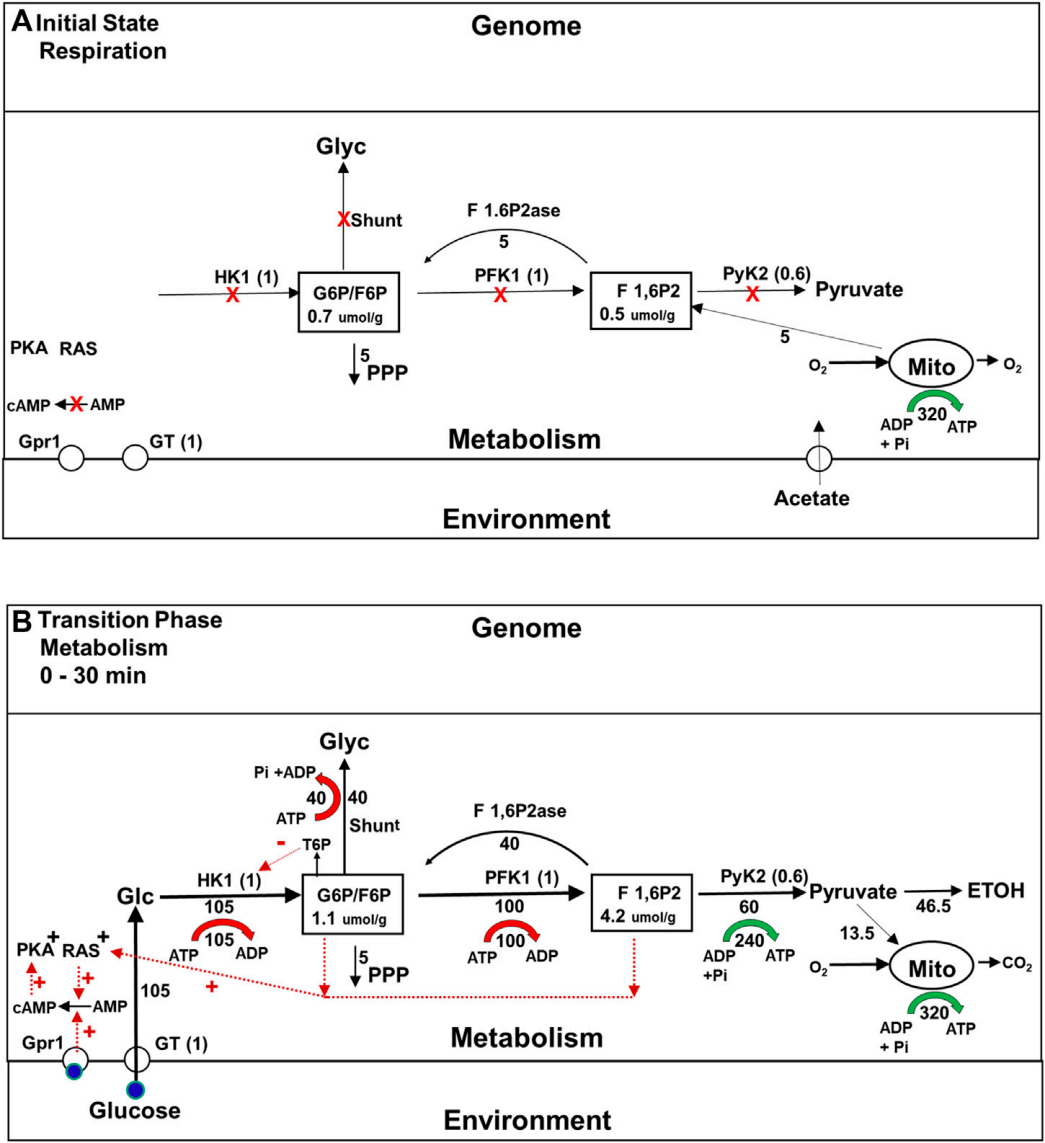
**(A,B)** Metabolic fluxes and intracellular signaling during the Initial State (Respiration) and initial Transition Phase (metabolism) during the Crabtree Effect. This figure provides information about the rates of key steps in the glycolytic, oxidative phosphorylation, and glycogen/trehalose shunt pathways, the concentrations of important glycolytic intermediates, and the isoforms of the enzymes that play the major role in flux and concentration control, including glucose transport. Maximum glycolytic enzyme activities are given in parentheses, normalized to their values during the final glycolysis state (Figure 4B). Metabolic fluxes are expressed relative to the flux through PFK in the final glycolytic state, which is set to 100 and indicated underneath or to the side the flux arrows. ATP synthesis and breakdown rates are shown as green and red arrows respectively, with the rates relative to PFK shown underneath. **(A)** Initial State (Respiration): When no glucose is present the activities of the enzymes in the glycolytic pathway are inhibited (red X) both because glycolytic intermediate concentrations are low and because glycolytic enzyme activities have been suppressed by post translational modifications. In addition signaling pathways that stimulate glucose metabolism, e.g., glucose- cAMP- PKA, and RAS, are downregulated. However the total concentrations of the glycolytic enzymes involved in producing F6P, e.g., HK1, PFK1, are the same as during the Final State of glycolysis. Below the intermediate F 1,6P2, enzyme concentrations are about 0.6 of final steady state values. G6P is produced by gluconeogenesis in order to support NADPH synthesis via the pentose phosphate pathway. Concentrations of G6P and other glycolytic intermediates are lower than when glucose is present. **(B)**Transitional Phase (Metabolism) Elevation of the glucose concentration leads to a rapid increase in the levels of intracellular glucose and phosphorylated glycolytic intermediates. Glucose binding to the Gpr1 receptor, and activation of the RAS signaling pathway in response to increased levels of glycolytic intermediates (red dotted arrows) activates the conversion of AMP to cyclic AMP, which in turn activates PKA as well as other signaling pathways. As a consequence of these interactions between signaling and metabolic pathways, the enzymes involved in glycolysis, as well as the glycogen shunt and futile cycling via F 1,6P2ase are proportionately activated. The flux through the initial portion of the glycolytic pathway rises to its maximum level, generating more substrate for the later part of the pathway than it is capable of processing. The excess is used to synthesize glycogen.

As the phenomenology discussed above makes clear, a lot more happens in the roughly 2 h it takes for yeast cells to become fully adapted to fermenting glucose than had been first thought. During the initial transition phase prior to gene expression changes, yeast rapidly adapt to the availability of glucose. The flux though their glycolytic enzymes increases both because there is now substrate available, and because they have been activated by post translational mechanisms that depend on allostery (Rothman et al., 2021) and enzyme modifications such as phosphorylations that are mediated by signaling pathways (Daran-Lapujade et al., 2007; Brink et al., 2018). However, it is not just the constitutive presence of glycolytic enzymes that endows yeast with the metabolic plasticity they require to survive a shift to a high glucose medium. In the early 1980s, studies of the metabolism of yeast mutants using ^13^C magnetic resonance spectroscopy (MRS) revealed that the glycogen and trehalose synthesis pathways, which we will refer to as the glycogen and trehalose shunts, and futile cycling pathways are very active early in the transition phase (see Figures 3A, B) (den Hollander et al., 1986a; den Hollander et al., 1986b; Shulman and Rothman, 2001). The importance of this fact was established by the mutational studies referred to above, but the requirement for these other pathways was surprising because it had long been thought that cells can control the flux through the glycolytic pathway, and other pathways simply by allosteric activation or inactivation of one or two rate limiting enzymes, as shown in Figure 2.

It would have been difficult to understand the role played by the shunts and futile cycling if the discipline called metabolic control analysis (MCA) had not come to maturity at about the same time. MCA originated in the mid 1970s, and since that time it has been a major contributor to the development of a rigorous, quantitative framework for understanding the properties of metabolic systems (Kascer and Burns, 1973; Fell, 1997). Prior to its development, the metabolic literature was littered with conclusions based on misconceptions about fundamental concepts having to do with the control of metabolic fluxes, e.g., the rate limiting step idea (see Fell, 1997).

Two of the several general theoretical and experimental principles discovered using MCA are relevant here. First, the flux through pathways is almost always controlled collectively by the activity and kinetic properties of many of the enzymes involved, not just one, and that is why mutations that alter the activity of PFK can have so little impact on the glycolytic flux (Davies and Brindle, 1992; Fell and Thomas, 1995). Second, MCA shows that branchpoint metabolites like glucose 6 phosphate (G6P) play a critical role in stabilizing metabolic systems. G6P is a substrate for glycogen synthesis, trehalose synthesis, the glycolytic pathway, and the pentose phosphate pathway, among others (see Figure 3B). As first shown by Kascer and Acerenza (1993), any change in activity of the enzymes in a pathway for which the branchpoint intermediate is a product, must be compensated for by a matching change in the activity of one or more of the pathways that use the branchpoint intermediate as a substrate. Otherwise the concentration of the branchpoint intermediate will change, and that will alter the flux through all of the pathways that intersect there, potentially leading to widespread metabolic dysregulation. It follows that homeostasis of branchpoint intermediate concentrations is critically important for the stability of metabolic systems (Hofmeyr, 2008; Hofmeyr and Rohwer, 2011). In the past 30 years, many mechanisms have been discovered that enable cells to proportionately increase or decrease the activities of enzyme involved in the production and consumption of branchpoint metabolites so that homeostasis can be maintained. They include allostery, enzyme phosphorylations and desphosphorylations triggered by signaling pathways, as well as changes in gene expression (Fell and Thomas, 1995; Shulman and Rothman, 1996; Hofmeyr and Rohwer, 2011). The importance of the glycogen and trehalose shunts in maintaining branchpoint intermediate homeostasis, which are side pathways that branch from G6P, has only more recently been realized (Van Heerden et al., 2013; Shulman and Rothman, 2015) and it is described below and in Figure 3B.

#### On the events that occur during the first transition phase of the Crabtree effect, plasticity conferred by the glycogen and trehalose shunts allows metabolic adaptation

Figure 3B shows during the initial transition phase the enzyme isoforms and transporters in the glycolytic pathway that exert the majority of control on the glycolytic flux, glucose transport (GT), hexokinase 1 (Hk1) and pyruvate kinase 2(PyK2). It also shows the critical shared branchpoint intermediates G6P and fructose 1,6, bisphosphate (F 1,6P2), and the glycogen shunt. In addition, two signaling pathways critical for proportional activation of metabolic enzymes are included: the glucose-cyclicAMP-phosphokinase A (PKA) pathway and the RAS pathway (Bendrioua et al., 2014; Peeters et al., 2017). When glucose is added to the medium, the activities of glycolytic enzymes and shunt enzymes rise for two reasons. First, the increase in intracellular glucose and, subsequently, G6P and other glycolytic intermediates allosterically activates glycolytic enzymes. Second, glucose binds to the membrane sensor protein Grp1 (illustrated) and others, and when it (blue circle) does so, the rate of synthesis of cyclic AMP (cAMP) by adenylate cyclase increases. The resulting increase in cAMP concentration activates PKA, and that, in turn, leads to the activation of glycolytic enzymes as well as glycogen and trehalose shunt enzymes (Peeters et al., 2017).

The role played by the shunts during the initial transition phase was not understood until quite recently. Using an advanced form of MCA called Supply and Demand Analysis (Hofmeyr and Rohwer, 2011), it was shown that the glycogen shunt stabilizes glycolysis by diverting to glycogen synthesis the portion of the flux of glucose to G6P and F1,6BP that is in excess of what the lower portion of the glycolytic pathway can utilize (Figure 3B) (Shulman and Rothman, 2015). In addition, the glycogen shunt and futile cycling at the phosphofructokinase 1 step (PFK1), which involves the gluconeogenic enzyme fructose bisphosphatase (F 1,6P2ase) together consume all the ATP synthesized glycolytically, which maintains homeostasis of energy charge (red and green arrows in Figure 3B). Teusink and colleagues (Teusink et al., 1998; Van Heerden et al., 2013) showed that under conditions in which yeast do not synthesize glycogen, homeostasis of G6P and F 1,6P2 are maintained by both feedback inhibition of HK1 by trehalose-6-phosphate (T6P) and ATP energy charge homeostasis by futile cycling between T6P and trehalose (not illustrated). Thus these studies demonstrated that proportional enzyme activation is critical for adaptation of glycolysis to the large shift in fuel supply, just as Kascer and Acerenza (1993) had predicted, but extended that theory by showing the importance of proportionate activation of several intersecting pathways.

#### On the events that occur during the second stage of the Crabtree effect transition phase, metabolism regulates adaptation of gene expression

**FIGURE 4:**
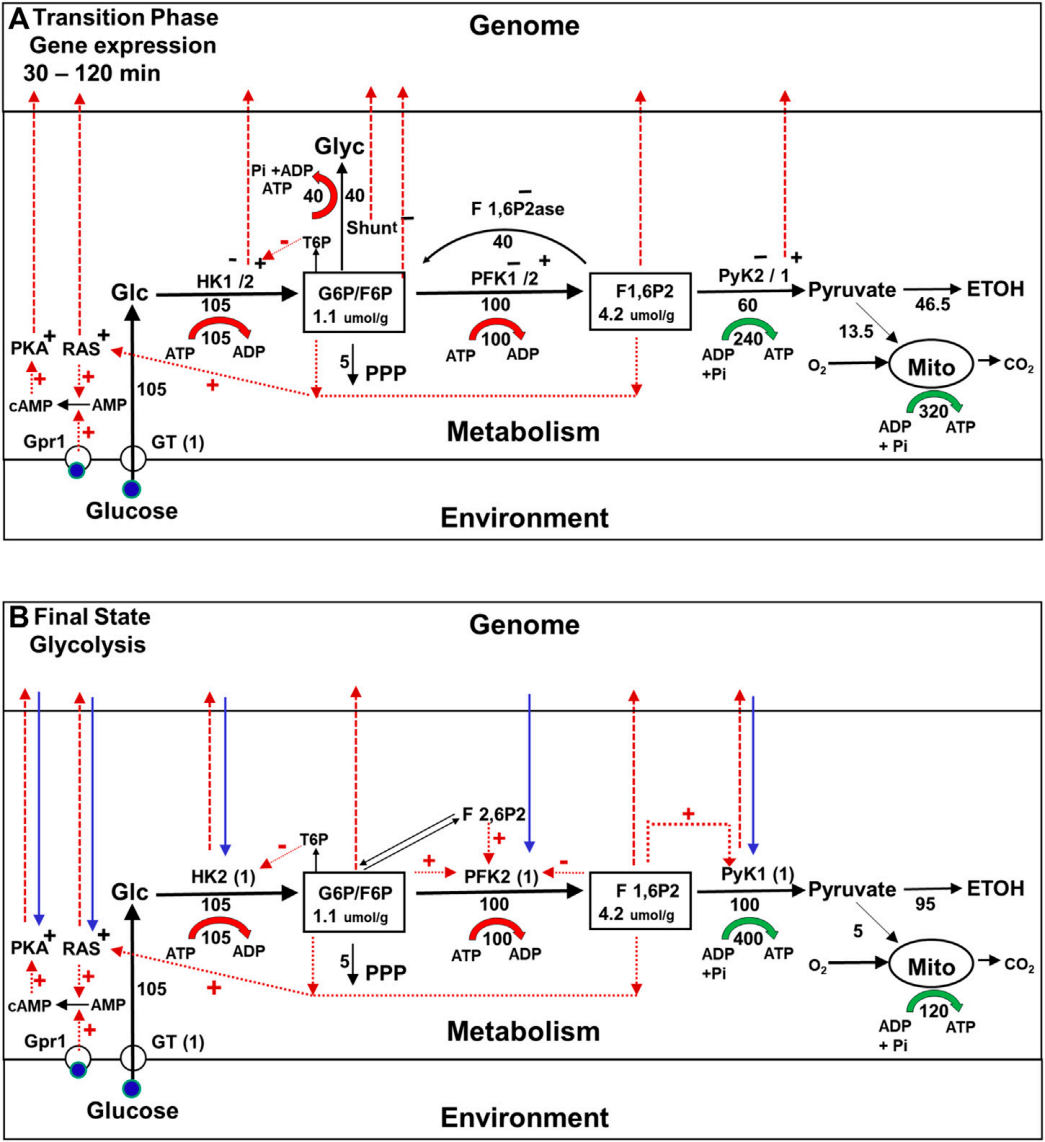
Metabolic fluxes, cell signaling, and gene expression during the Crabtree Effect: second stage of the transition (gene expression) and final state (glycolysis). **(A)** Second stage of the transition phase (Gene expression). The first stage of the transition phase ends about 30 min after the glucose challenge when the metabolic system reaches steady state. The enzyme isoforms present at this time are represented as HK1^−^/2^+^ PFK1^−^/2^+^, PyK2^−^/1^+^, with the minus sign indicating an isoform that is no longer being expressed and the + sign indicating an isoform the synthesis of which has been activated. The red dashed lines from HK2, PyK1, and Shunt to the genome indicate that the enzyme indicated is being translocated to the nucleus where it will form complexes that alter the expression of genes for many proteins including themselves. The dashed red lines to the genome from G6P, F6P, and F 1,6P2 indicate that these glycolytic intermediates are also involved in activating and/or inhibiting gene expression. In addition, cellular signaling pathways, e.g., the glucose-cAMP-PKA, and RAS pathways, which were activated during the first stage of the transition phase now contribute to the control of gene expression in the nucleus. **(B)** Final State (Glycolysis) Approximately 120 min after glucose addition, the cells reach a new steady state they can sustain for extended time periods. The glycolytic enzyme isoforms present initially, e.g., HK1, PFK1, and PyK2, have been replaced by new isoforms, e.g., HK2, PFK2, and PyK1 (blue dashed arrows). The synthesis enzymes in the glycogen shunt, as well as F 1,6P2ase, are repressed. Regulatory interactions between the metabolism and gene expression and *vice versa* (red and blue arrows) ensure the stability of this new regime.

After approximately 30 min, a new metabolic steady state is achieved, as shown in Figure 3B, after which changes in gene expression commence (Figure 4A). As pointed out earlier, they result in wholesale replacement of isoforms in the glycolytic pathway, as well as in other pathways, and in regulatory pathways such as the RAS and glucose-cAMP-PKA pathways. At the same time, the enzymes in the glycogen and trehalose synthesis pathways are almost completely suppressed. Interestingly, these changes in levels of gene expression are not caused directly by the change in the external concentration of glucose that triggers the onset of the Crabtree effect. In addition, they do not alter the rate at which glucose is being consumed.

The reason these enzymatic alterations have selective value is that the shunts consume ATP that can be better used for other purposes. Futile cycling and glycogen synthesis, which are necessary for maintaining homeostasis during the first stage of the transition state, come at a cost. When yeast cells are operating this way, glycolysis results in no net ATP production. In addition, these alterations allow all of the glycogen synthesis flux to be used for ethanol production, which is believed to provide yeast species that exhibit the Crabtree effect a competitive advantage over other microorganisms (Pfeiffer and Morley, 2014; Hagman and Priskur, 2015).

But how does the cell suppress the shunts while still maintaining homeostasis of branchpoint intermediates, and why is a whole sale replacement of glycolytic enzyme isoforms required? Using supply and demand MCA (Hofmeyr and Rohwer, 2011), we recently showed that if there was no change in glycolytic enzyme isoforms, once the shunts were suppressed the glycolytic pathway would be unable to maintain branchpoint intermediate homeostasis as shown in Supplementary Figure S1 (Rothman et al., 2021). The main difference between the new and original isoforms, is that the new isoforms are much more sensitive to allosteric activation and inhibition both by glycolytic intermediates, and by intermediates in the coupled fructose 2,6 bis phosphate (F 2,6P2) pathway (Figure 4A). As a result, this version of the glycolytic pathway can maintain branchpoint intermediate homeostasis by itself (Rothman et al., 2021; see Supplementary Figure S1). Consequently, the shunts can be, and are inactivated, and the organism benefits because their suppression increases the net production of ATP per ethanol molecule produced, as well as increasing the amount of ethanol produced per glucose consumed.

For those familiar with the view that gene expression drives metabolic adaptation, it may come as a surprise that the changes in gene expression that occur during second stage of the Crabtree effect transition phase are not induced directly by the change in external glucose concentration. As shown by the red arrows in Figure 4A, changes in gene expression are instead activated by many mechanisms, all of which are coupled to metabolism either directly or indirectly. Among these mechanisms are some that depend on complexes in the nucleus that are activated by increases in the steady state concentrations of the glycolytic intermediates from during the first stage of the transition phase (Lorendau et al., 2015), and complexes formed by translocation of specific glycolytic enzyme into the nucleus (Van der Knapp and Verrijzer, 2016; Kochanowski et al., 2017; Li et al., 2018; Wang and Li, 2018). Even the glucose-cAMP-PKA pathway, which was originally thought to couple changes in glucose concentration directly to gene expression, requires metabolic adaptation to be activated. For example, as illustrated in Figure 4, activation of the pathway depends on an increase in cAMP that is synthesized by adenylate cyclase, the activity of which depends on increases in initial transition phase the concentrations of intracellular glucose and of glycolytic intermediates that activate yet another signaling pathway (RAS) (Peeters et al., 2017; Kunkel et al., 2019) (see Figure 4A, dotted red lines). Similar mechanisms have been identified in mammalian cells, including the ChREBP_alpha_ pathway, which regulates transcription of enzymes in the glycolytic and lipogenesis pathways in response to carbohydrate feeding and is directly regulated by G6P and F 2,6P2 and pentose phosphate pathway intermediate levels (Abdul-Wahed et al., 2017). Thus, in adaptation of yeast to environmental glucose in the Crabtree effect, one could make the case that it is metabolism that is controlling gene expression rather than the other way around.

#### History dependence of the regulation of metabolism and gene expression during the final state of glycolysis

After approximately 120 min a new steady state is reached, in which the flux through the glycogen shunt is repressed, and the glycolytic enzymes that were present initially have largely been replaced by new isoforms (blue dashed arrows in Figure 4B), as have the isoforms of many other many other metabolic enzymes and proteins in signalling pathways. It is important to point out that the feedback between metabolic pathways and the gene expression system that made these isoform replacements possible continues indefinitely, as shown by the reciprocal red an blue arrows in the figure. Thus the new steady state is stabilized by a control system that has both genetic and metabolic components, as is also the case for the initial steady state.

It is also important to point out that the properties the cells display after the genetic portion of the adaption process has run its course are not determined solely by the new environmental conditions, as one might expect if adaptation was entirely driven by genetics. They depend on the history of the cells before it was challenged by the addition of glucose to the medium, and that is true also for the process that unfolds during the transition phase. For example, depending on the amount of glycogen the cells contained when they were first challenged by the addition of glucose to the medium, as well as the presence or absence of other substrates such as amino acids, the net effect of the glycogen shunt will either be to synthesize glycogen, or to break it down, or engage in trehalose futile cycling (Van Heerden et al., 2013; Rothman et al., 2021). Longer term, the life history of transitions between fermentation and respiration have been shown to influence how yeast adapt to changes in carbon sources (Cerulus et al., 2018). As described in the Section 2, there are many post translational and epigenetic mechanisms that enable cells to “remember” their prior histories, independent of the specific gene products that may be present.

### Transition phase description of the lac operon

#### The lac operon, the 1960s model for the adaptation of *E. coli* to growth on lactose

By around the 1960s, it was known that *E. coli* must express the genes in its lac operon at a high level if it is to grow on lactose. The two most important proteins encoded by that operon are lac permease, the membrane protein that facilitates the entry of lactose into the cell, and beta galactosidase, the enzyme that catalyzes the hydrolysis of lactose to glucose and galactose, which is the first step in lactose metabolism. It was further understood that when glucose is available to *E. coli* cells, expression of the genes in the lac operon is suppressed by the binding of a protein called the lac repressor to a sequence at the upstream end of that operon. The affinity of the lac repressor for the lac operator is dramatically reduced when any one of several different small molecules called inducers bind to it, and the rate at which the genes in the lac operon are expressed is several hundred-fold higher when there is no repressor bound than it is when there is repressor bound. Not long after Jacob and Monod (1961) described this system, the inducer for the lac operon that is effective *in vivo* was identified. It is allolactose, an isomer of lactose (Muller-Hill et al., 1964) that is the product of a side reaction that occurs when beta galactosidase catalyzes lactose hydrolysis (Huber et al., 1976).

**FIGURE 5:**
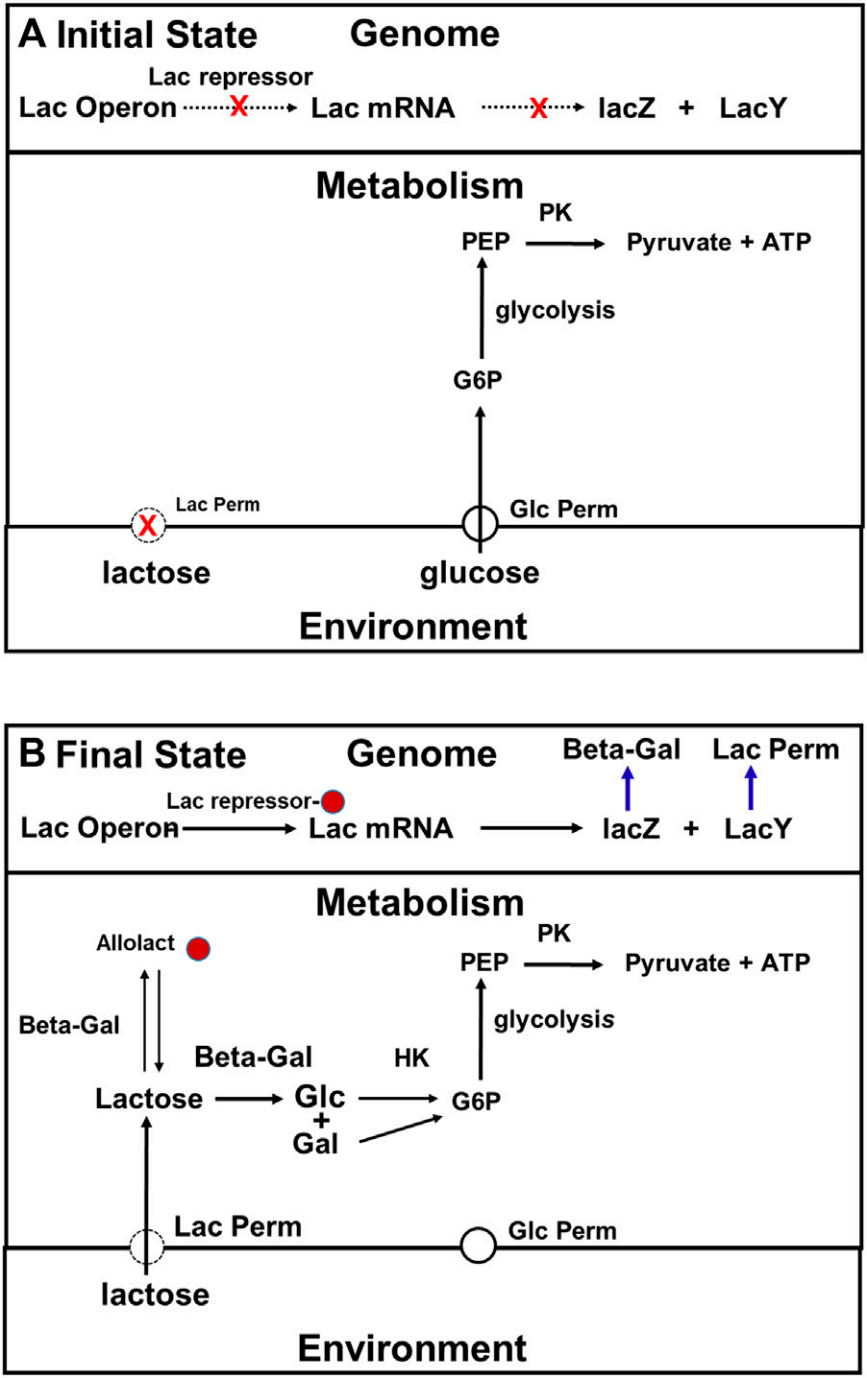
The traditional, two-state, top-down description of the adaptation of E. coli to growth on lactose. **(A)**: When both lactose and glucose are present in the medium, cells use glucose rather than the lactose because the number of lac permease and beta galactosidase molecules present per cell is maintained at a low level both because expression of the lac operon is being inhibited by the repressor, the total cell volume in the culture is growing rapidly. **(B)**: The repressor is in its inactive state because it has inducer bound. Consequently, the lac operon is being expressed at maximum rate, and the numbers of lac permease and beta galactosidase molecules present are high enough to support log phase growth on lactose. Abbreviations: G6P, glucose 6 phosphate; PEP, phosphoenolpyruvate; Pyr, pyruvate; Lac permease, lactose permease; Beta -Gal (beta-galactosidase); PK, pyruvate kinase; HK, hexokinase.

By the mid-1960s, the model for how cells that have been growing on a medium that contains both glucose and lactose adapt after the glucose runs was quite simple (Figure 5). Initially, when both glucose and lactose are available, they use glucose as their carbon source rather than lactose because expression of the lac operon is being inhibited by the repressor. In addition, as in the Crabtree effect, the glycolytic and other enzymes related to glucose metabolism are synthesized constitutively in *E. coli* (Buffing et al., 2018). It is important to point out that lac permease and beta galactosidase are synthesized at a low rate under the conditions just described, and, therefore that lactose is constantly entering these cells, and as a consequence of its being metabolized, allolactose is constantly being produced. The reason the number of molecules per cell of lac permease remains low is that rate at which lac permease is being synthesized is modest compared to the rate at which it is being diluted by cell growth. Because the number of lac permease molecules per cell is low, the concentration of lactose in these cell will remain low both because of its rate of entry into these cells is low, and because it is constantly being metabolized, and diluted by cell growth (see Yildirim and Mackey, 2003). If the intracellular concentration of lactose is low, the allolactose concentration will be low also.

When the glucose supply is exhausted, growth stops, and dilution stops also. The concentrations of both lactose and allolactose in the cytoplasm begin to rise. At some point, the allolactose concentration gets high enough so that the inhibition of the lac operon by the repressor begins to be relieved. The rates of synthesis of lac permease and beta galactosidase will start to rise, which lead to a further increase in allolactose concentration, further favoring the derepressed state of the lac operon. Once the amounts of both enzymes get high enough, the cells will be able to grow on lactose. Thus, as far as anyone knew in the early 1960s, the adaptation just described depended entirely on the properties of the repressor and the proteins encoded by the lac operon, and that beyond keeping the cells alive, the metabolism that goes on in the cell during the ∼45 min it takes for cells to switch from growth on glucose to growth on lactose was irrelevant.

#### The role played by metabolism during the transition phase in regulating transcription of the lac operon

As already noted, the transition phase that is such an obvious feature of diauxic growth was neglected in the original model for the glucose to lactose adaptation in *E. coli*, and that neglect is evident in textbook descriptions of the control of the lac operon, and even in recent models for this phenomenon that take epigenetic modifications into account (see Section 2). This is surprising because it ignores an important problem that bacteria undergoing this adaptation cannot: where is the supply of energy and metabolites they need to survive, and ultimately to synthesize lac permease and beta galactosidase to come from once they run out of glucose? Fortunately, over the past 60 years, a lot has been learned about what happens during the 45 min transition phase of the glucose to lactose adaptation, and we now know that even this adaptation is not entirely top-down.

**FIGURE 6:**
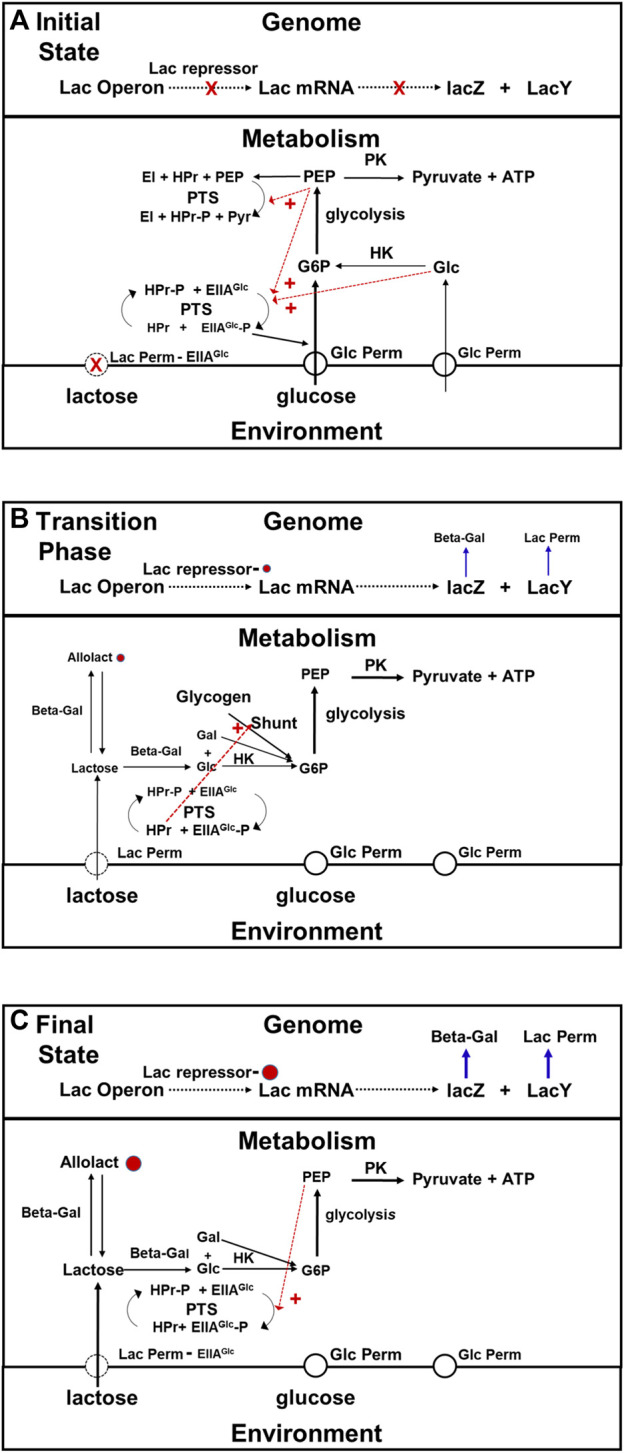
Transition phase model for the adaptation that occurs when *E. coli* switch from growth on glucose to growth on lactose. In this figure black arrows represent metabolic pathways, blue arrows represent transcription and gene expression, and dotted red arrows represent allosteric activation and inhibition, **(A)** Initial state of glucose metabolism. When glucose is present in the medium, *E. coli* uses it in preference to alternate substrates. The rate at which lactose is being taken up from the medium is very low because there is very little lac permease being synthesized, and what little there is being inhibited by ELLA^Glc^, which is the dominant form when intracellular glucose and PEP concentrations are high (red dashed arrows) due to activation of phosphate transfer to glucose permease via allosteric activation by PEP and intracellular glucose. Under these conditions, the combination of inhibition of lactose transport, dilution caused by growth, and residual metabolism maintain the intracellular concentration of lactose at a very low level (see text). As a consequence, there is little allolactose present and the lac repressor protein is bound to the operon, blocking transcription. **(B)** Transition Phase (Metabolism). Exhaustion of the glucose supply relieves the inhibition of lactose permease, due to the conversion by the PTS system of EIIA^Glc^ to EIIA^Glc^–P in response to the drop in intracellular glucose and PEP concentrations. The cessation of growth brings to a halt intracellular lactose dilution. The intracellular concentrations of both lactose and allolactose begin to rise. During this, phase, the cell obtains the energy and precursors in needs via the glycogen shunt, which itself is activated by proteins in the PTS system. **(C)** Transition Phase (Gene Expression) and Steady State Lactose Metabolism. Not long after the metabolic transition phase begins, the concentration of allolactose rises enough to begin inactivating the repressor. After approximately 45 min, growth on lactose will begin, and the cells will enter a new steady state in which rates of gene expression and rates of production of metabolites are balanced by rates of consumption and rates of dilution due to cell growth. As the rate of lactose metabolism rises, the intracellular glucose and PEP concentrations will begin to recover. This will result in a decrease in the activity of the lactose permease due to a drop in EIIA^Glc^–P and increase in EIIA^Glc^ levels. As a consequence, a balance between allolactose synthesis and breakdown is achieved and the rate of transcription reaches steady state. Abbreviations: G6P, glucose 6 phosphate; PEP, phosphoenolpyruvate; Pyr, pyruvate; Lac permease, lactose permease; Beta -Gal, beta-galactosidase; PK, pyruvate kinase; HK, hexokinase, shunt, glycogen shunt; CAP, catabolite activator protein; EI, enzyme I of the phosphotransferase system; EIIA^Glc^, the A subunit of enzyme II of the phosphotransferase system; EIIA^Glc^ -P, phosphorylated A subunit of enzyme II of the phosphotransferase system; HPr, phosphocarrier protein of the phosphoryl tranferase system; PTS, phosphotranferase system.

The key actor in this drama that Jacob and Monod did not know about in 1960 is EIIA^Glc^, (see Figure 6). EIIA^Glc^ is a component of the phosphotransferase system (PTS) that is responsible for the active transport of glucose into *E. coli* cells (for reviews see Kotrba et al., 2001; Escalante et al., 2012). The PTS system catalyzes the transfer of a phosphate to EIIA^Glc^ originating from PEP, via intermediate steps using the EI and HPr proteins (see Figure 6). When glucose metabolism is active, the phosphate group on EIIA^Glc^ -P, is rapidly transferred to glucose entering via the glucose permease. G6P is the product. When cells are actively metabolizing glucose, the concentration of unphosphorylated EIIAGLlc exceeds that of its phosphorylated form. Unphosphorylated EIIA^Glc^ binds to the lactose permease inhibiting it. Thus, when there is glucose available, not only will the number of lac permease molecules per cell be small, but their activities will be lower than they would have been otherwise due to their interactions with EIIA^Glc^. When the supply of glucose become depleted, the fraction of the EIIA^Glc^ molecule present that are phosphorylated will rise because the rate of glucose transport has fallen, and the rate at which lactose enters cells will increase because the fraction of the lac permease molecules inhibited by EIIA^Glc^ has decreased. The intracellular concentration of allolactose will rise in response, increasing the probability that the repressor protein is bound and therefore the expression of the lac operon be derepressed. A secondary component in this regulatory drama is CAP, a protein that binds cyclic AMP (cAMP). When cAMP is bound to it, it binds with high affinity to a specific site at the upstream, promoter end of the lac operon. Prior to the working out of the PTS, it was believed that CAP played a key supplementary role in the regulation of gene expression along with lactose and allolactose (see Saier, 1989; Busby and Ebright, 1999). However, more recent studies have shown that CAP has a minimal role in regulation of lac operon transcription (Inada et al., 1996) other than under special conditions such as providing additional repression during cell cycle perturbations (Vilar and Saiz, 2023).

Thus the state of phosphorylation of EIIA^Glc^, which depends on the metabolic state of the cell, controls the rate of expression of the lac operon system in two entirely different ways: 1) by affecting the concentration of allolactose present, and 2) by changing the efficiency with which the lac operon is transcribed. The lac operon is *not* autonomously regulated. Its regulation is mediated by the cell’s metabolic system. It is bottom-up.

The transition phase for this adaptation. Begins when the glucose supply runs out, and growth ceases. During the first stage of the transition, three things happen that will ultimately result in the de-repression of the lac operon: 1) the dilution caused by growth stops, 2) the inhibition of lac permease by EIIA^Glc^ ceases, and 3) the concentration of cAMP rises because the increase in the concentration of the phosphorylated form of EIIA^Glc^ activates adenyl cyclcase This system displays positive feedback (Aggarwal and Narang, 2022). As soon as amounts of permease and beta galactosidase get high enough, the cells will be able to sustain themselves by metabolizing lactose, and growth will resume. However until that time comes, the cells must support themselves by consuming materials they already contain, most notably glycogen (see Figure 6B). The glycogen shunt operates in reverse (glycogen breakdown), and that produces the G6P the cell need both for ATP production, and for the synthesis of other metabolites until expression of the enzymes required for lactose metabolism reaches steady state. It is upregulated when the concentration of EIIA^Glc^ is low, and concentration of cAMP is high, as a result of low glucose levels (Tian et al., 2013). In addition to these functions, the EIIA^Glc^ system regulates the activity of several other proteins involved in glucose uptake, metabolism, and repression of transcription, which is equivalent to the proportional activation and deactivation described for the Crabtree effect (Deutcher et al., 2006; Börke and Stülke, 2008; Bao and Duong, 2013). This is metabolic plasticity at work.

Note that when growth resumes, growth-related dilution will again come into play, and the concentrations of all of the molecule involved in lactose metabolism will be determined by the ratios of their rates of synthesis/uptake to their rates of dilution/destruction. It is important to realize that the mechanisms that repressed lactose expression during the initial state, and were not operational during the transition phase, play a role in determining the characteristics of the new steady state. They are activated because the hydrolysis of lactose catalyzed by beta galactosidese generates glucose directly, as well as galactose, which is ultimately converted to glucose. Therefore, even in the final state, metabolism is still having an effect on gene expression.

It is important to point out that the rate of gene expression in the final state depends not only upon initial environmental conditions, but also the cell’s prior history (Santillan and Mackey, 2008; Lambert and Kussel, 2014). Most of the work done in this area has focused on epigenetic mechanisms that affect the activities of lactose permease and beta galactosidase at the beginning of the transition phase, and have modeled their impact on the upregulation of operon transcription. However, as discussed below, given the strength of the regulatory interactions between glucose and lactose metabolism, it is possible that history-dependent variations in the activities of enzymes involved may be just an important. The finding that in fluctuating environments the operon transcriptional response to lactose depends on the starting levels of proteins involved in lactose metabolism supports this assertion (Lambert and Kussel, 2014). Furthermore, it now appears that many metabolites contribute to the control gene expression in *E. coli* (Lempp et al., 2019).

The importance of metabolic plasticity in a transition state, and how successful long term adaptation depends upon it, is demonstrated by the phenomenon referred to as lactose killing (Dzykhuizen and Hartl, 1978). In this phenomenon, *E. coli* cells adapted to lactose metabolism are switched to a minimum medium with no other carbon, nitrogen, and magnesium and other factors needed for growth. Surprisingly, 80%–98% of the *E. coli* cells die, as a result of excessive influx of lactose through the lactose permease (Dykuizen and Hartl, 1978). We suggest that the lethal increased flux through the lactose permease is a consequence of the EIIA^Glc^ system, which integrates information from multiple substrates and metabolic pathways in the cell, not having the necessary inputs to inhibit lactose permease. It is an example of the case in which the metabolic plasticity of the initial transition state is insufficient for the cell to survive the environmental change.

## Discussion

### On the history dependence of final states

Since the time of Mendel, if not before, biologists have realized that organisms have long term memory systems, i.e., genomes, and by the middle of the 20th century, it was realized that they are made of nucleic acid. The sequence information in an organism’s genome is remarkably stable, and largely insensitive to changes in its environment or to its lifetime experiences. Genomic changes, i.e., mutations and/or rearrangements, are rare, and largely random events, but they can have consequences that affect the fitness of an organism and its progeny into the indefinite future. If it were true of all metabolic adaptations that the physiological properties of cells that have undergone any particular metabolic adaptation were independent of the properties they displayed in their initial steady states, and that they could be accurately predicted using genomic information alone, the contributions made by metabolism to adaptations, however interesting they might be, would be of mechanistic interest only. The metabolically determined vs. genetically determined distinction made earlier would then be a chimera because it would then be possible to argue that outcomes are 100% genetically determined.

The issue of whether genetics fully explains all adaptations was addressed in the summary paper Jacob and Monod wrote for the 1961 Cold Spring Harbor Symposium in Quantitative Biology (Monod and Jacob, 1961). Many examples of adaptations were discussed at that meeting that seemed consistent with the genetically driven scheme they had developed for the lac system, but there were several that seemed to require that cells “remember” what their previous environments were like, which seemed “ungenetic” (e.g., Davis, 1961). There were still many advocates of the view, championed by Delbruk (1949) and colleagues, that there are mechanisms that enable the cell to retain information about its history, independent of its genes. Today we know about many epigenetic and post translational mechanisms that do just that. However in 1961, as Monod and Jacob pointed out in their article, allosteric induced changes in protein conformation were the only molecular alterations other than genetic mutation then known, that could conceivably endow cells with memory-like properties. However, the lifetimes of conformational states is far too short for them to have an impact on minutes to hours time scales. In an effort to explain how the memory effects their colleagues had uncovered might work, they pointed out that pairs of interacting operon/repressor systems could function like electronic flip-flop circuits, an observation that inspired the development of operon-like modules for biological computers 40 years later (Hengen et al., 2003). The quote below explains what they had in mind:

“. . for instance, in the system shown below (Figure 5) a regulator gene controls the synthesis of enzymes within an operon which includes another regulator gene acting upon the operator to which the first one is attached. Such a system would be completely independent of the actual metabolic activity of the enzymes and could be switched from the inactive to the active state by transient contact with a specific inducer, produced for instance only by another tissue”

Systems like these could make cells “remember” what the conditions were that they had experienced earlier, but would be genetic in nature, rather than metabolic.

Since the early 1960s, there has been a tremendous increase in our knowledge of non genetic mechanisms that make the responses of cells to environmental challenges dependent on their prior histories. We have alluded to only a small fraction of them here, e.g., see (Riber and Hansen, 2021). Futhermore, as pointed out above, they modulate the responses of yeast experiencing the Crabtree effect, and they have a similar effect on the lac operon diauxic phenomenon in *E. coli*.

### The role of metabolism in regulating post translational modifications

Although epigenetics is still considered by many to be the major mechanism that makes cellular adaptations history-dependent, recent studies have revealed that post translational modifications of proteins and cellular organelles can have the same effect. For example, long term conditioning of muscle mitochondrial metabolism by exercise has been shown to be regulated independently of both gene expression and epigenetic modifications of the genome (Stolle et al., 2018). The post translational modifications that result in this adaptation, are clearly metabolically driven.

As shown in Figure 4, any time a yeast cell is exposed to high glucose concentrations in its medium there will be post translational modifications made to its metabolic enzymes that are mediated by signaling pathways, and to the proteins in the signaling pathways themselves. Conversely, the activity of signaling pathways are determined by the metabolic state of the cell, not just the presence of glucose in the environment. Although only the response to glucose is illustrated, metabolic pathways of other substrates, such as amino acids, also strongly interact with signaling pathways, as well as directly with glucose metabolism. These changes can have long lasting effects on the properties not only of the enzymes involved in glycolysis and the shunts, but also on those of a large fraction of the cell’s other enzymes and signaling proteins, phosphorylations/dephosphorylations being the primary mechanism (Oliveira et al., 2012; Oliveira and Sauer, 2012; Vlastaridis et al., 2017). The widespread nature of these post translational modifications of metabolic proteins is consistent the findings made using MCA, which have shown that proportional activation of enzymes in many pathways are likely to be required to control both fluxes and branchpoint intermediate concentrations. Although changes in enzyme phosphorylation have traditionally been thought of as a means to control flux (Chen and Nielsen, 2016), we and others have shown that it also plays a critical role in maintaining branch point intermediate homeostasis (Shulman and Rothman, 1996; Fell and Thomas, 1995; Palm et al., 2012; Schafer et al., 2004; Nozaki et al., 2020). The discovery that disruption of individual kinases and phosphatases can have widespread impact on fluxes and intermediate concentrations in yeast is consistent with this point of view (Schulz et al., 2014).

### The role of metabolism in regulating epigenetic modifications

Epigenetic modifications are often thought of as direct responses to environmental changes that are not mediated by metabolism. For example, in *E. coli*, lactose activates systems that produce long lasting alterations in lac operon activity by acetylating chromatin (Santillan and Mackey, 2008). It is easy to overlook the role that metabolism might play in phenomena like these, and that neglect makes it easy to incorporate epigenetic mechanisms neatly into genetically driven models for metabolic adaptation (Tzika et al., 2018). However, recent studies have shown that in both eukaryotes and prokaryotes, epigenetic mechanisms that affect the expression of genes that encode metabolic enzymes are regulated by metabolism (Ward and Thompson, 2012; Kinnaird et al., 2016; Etchegaray and Mostoslavsky, 2016; Carthew, 2020). It is time that across a wider range of fields, models for the epigenetic regulation of gene expression take account of the role played by metabolism, just as it is time that its role in regulating gene expression during metabolic adaptation be taken into account also.

### What are the advantages of having a metabolic transition phase that also regulates changes in gene expression?

We can think of several reasons why organisms might have evolved to have a high degree of metabolic plasticity, and to use metabolism during the transition phase to regulate gene expression as well as alter the properties of cells over the long term epigenetically, and by post translational modification of proteins. The most obvious of them is speed. Adaptations that depend on changes in gene expression are slow to occur. Even in fast-growing organisms like bacteria, they can take tens of minutes to become manifest. The processes that modulate enzyme activities have much faster time constants, a second or better. The Crabtree effect provides a case in point.

There is also a more subtle benefit to having metabolism contribute to the control of gene expression. With the development of techniques or measuring gene expression in single cells, it has been discovered that for stochastic reasons, within any given population, there can considerable, cell-to-cell variation in the capacity to respond to environmental stimuli. These are caused variations in protein levels in the initial state (Van Heerden et al., 2013; Bhogate et al., 2014; Lambert and Kussel, 2014). There is no way the genome can respond to these variations unless feedback and feed forward systems exist that will enable the metabolic state of the organism to play an active role in determining levels of gene expression. For example, our metabolic modeling of the Crabtree effect suggested that variations in the activity of glycolytic enzymes relative to the glycogen shunt could have a significant impact on the metabolic transition state (and its stability) (Rothman et al., 2021), which is in agreement with experimental findings (Van Heerden et al., 2013).

When thinking about the way metabolism interacts with the genome, one is led to wonder how much information about the metabolic state of an organism needs to be stored and processed by the systems that regulate gene expression. This issue was first commented upon by scientists like Schroedinger (1944), and Delbruck (1949), and as described above, it was an important issue in early debates about the control of adaptation (Monod and Jacob, 1961). If the systems regulating gene expression monitored every aspect of the physiological state of an organism, they would effectively possess all the information needed to predict the metabolic response of that organism to any changes whatever in the environment or in levels of gene expression. In principle, an organism that contained an internal information system that comprehensive would be able to manage its metabolic adaptations by responding directly to the environment the way the lac operon system was originally thought to do. However, as Hofmeyr (2008); Hofmeyr and Rohwer (2011) have suggested, the metabolic system automatically does much of the work such a hypothetical control system would have to do. As a result of the supply and demand structure of metabolic the activities of a comparatively small number of enzymes control both pathway fluxes and branchpoint intermediate concentrations. For example, most of what happens during the Crabtree effect can be predicted if you know the activities of specific isoforms of glucose transporters, hexokinase, the glycogen shunt, and pyruvate kinase, and you are tracking the concentrations of G6P and F 1,6P2 (see Figure 3B). Consistent with this proposal, Chubukov et al. (2012) have shown that the expression of the genes that encode metabolic enzymes in yeast is strongly influenced by a phenomenon they refer to as intermediate metabolite activation, and therefore a relative small number of metabolites and enzymes are critical for the regulation of metabolism. Similar findings have been reported for central metabolism in *E. coli* (Kochanowski et al., 2017). These findings were anticipated by the earlier work done using MCA by Kascer and Acerenza (1993), and much earlier than that by Davis (1961), in the same issue of Cold Spring Harbor Symposium in Quantitative Biology as the Monod and Jacob paper quoted from above (1961). (NB: Supply and demand control points can change as the state of an organism changes. For example, the glycogen shunt has a key role in controlling G6P and F 1,6P2 concentrations during the initial transition phase in Crabtree yeast, but is almost completely suppressed during steady state fermentation during which new glycolytic isoforms take over this homeostatic function).

### A potential role for metabolic plasticity during evolution

The high metabolic plasticity evident during the transition phases of the two adaptations described above may play a role in the much longer term adaptations that occur during evolution. The majority of research in evolutionary biology follows what has been called the Modern Synthesis (Mayr, 1982). This synthesis is a genetically driven paradigm, in which, on a molecular level, long term adaptation is almost completely explained by genetic mutations or other structural modifications (Smocovitis, 1992). However, some have pointed out that natural selection also depends on the ability of organisms to adapt to environmental changes well enough to survive, long before the genetic changes occur that will ultimately improve their fitness under the new conditions. They refer to this property of organisms as phenotypic plasticity (West-Eberhard, 2005; Bateson, 2014; Fox et al., 2019). Their argument is that, absent the requisite phenotypic plasticity, a population of organisms may not survive an environmental change long enough for natural selection to occur.

Proponents of the phenotypic plasticity concept have primary identified the source of this plasticity as being determined by the range of gene expression an organism is capable of, which may be expanded epigenetically (West-Eberhard, 2005; Bateson, 2014; Sommer, 2020). We propose that this view be extended to include the mechanisms that confer metabolic plasticity during the initial transition phase. As described for the Crabtree effect, and for *E. coli* during the transition to lactose metabolism, the organism would not survive long enough for adaptation by changes in gene expression to occur if the glycogen shunt was not available to either consume excess G6P (yeast), or to provide G6P for ATP production while transcription of the lac operon is being upregulated (*E. coli*). Recently, mathematical simulation led us to conclude that if ancestral organisms did not have the metabolic plasticity provided by the glycogen shunt, the evolution of the Crabtree effect would not have occurred because mutations that led to increased activity of glycolytic enzymes proximal to F 1,6P2 would result in excessive increases in glycolytic intermediates as well as depletion of ATP levels that the cells could not survive (Rothman et al., 2021). We advocate that those working on the evolution of metabolic phenotypes incorporate metabolic plasticity into their models.

### Is it possible to determine the relative contributions to the control of adaptation made by metabolism, gene expression, and signaling pathways?

Analysis of the adaptations phases for the Crabtree effect and *E coli* lactose metabolism demonstrates that neither the traditional views of genetic driven control (Desvergone et al., 2006), nor more recent views of signaling pathway (Ward and Thompson, 2012; Magaway et al., 2019), epigenetic (Tzika et al., 2018), and metabolic driven control (Lorendau et al., 2015) can fully describe the complexity of what happens during metabolic adaptations. Therefore, it is essential that methods be developed for analyzing adaptations that can integrate the effects of all of these interdependent mechanisms. Using MCA as a guide, it may be possible to integrate metabolism into current models of genetic, epigenetic, and signaling pathway regulation and arrive at MCA-like quantitative definitions of control that are applicable to short and long term metabolic adaptation. In fact several recent publications have integrated MCA and genomic analyses in microorganisms with good agreement with experimental findings (e.g., Lieven et al., 2020; Pearcy et al., 2022). However, it appears likely to us that the prevalent idea that adaptation is primarly controlled by a single mechanism, or even a single level of mechanisms (e.g., genomic, metabolic) will have to be given up along the way.

## Conclusion

Given the failure of models for metabolic adaptation that are either purely genetic or purely metabolic to fully describe even the simplest examples of metabolic adaptation, it seems to us that it is high time to stop trying to generate models of either type. Neither should be pursued to the exclusion of the other either in the introductions and/or discussions of papers, let alone in educational materials such as text books. Instead, explanations of adaptive processes should acknowledge the roles played by metabolic phenotype and plasticity as well as changes in gene expression. Although the models for adaptation that emerge will be more complex than they would be otherwise, they will force both investigators and students to confront the dynamic interactions that occur between the environment, a cell’s metabolism, and its genome, the understanding of which is the true challenge for those concerned with adaptive processes in biology. A similar conclusion applies to the integration of signaling pathways and epigenetic and post translational modifications.

One of the goals of this paper is to call attention to the importance of metabolic plasticity for both short-term and long term metabolic adaptation. Despite the advances that have been made in identifying metabolic mechanisms such as the glycogen and trehalose shunts that confer plasticity, and the many direct feedback and feed forward control mechanisms that make it possible for metabolic systems to influence gene expression, the number of publications that include metabolism in their final descriptions of adaptive control is small. Perhaps even more regrettable, the dynamic interplay between the environment, metabolism, signaling pathways, and gene expression that occurs during transition phases is largely omitted. Instead, no matter how complex and interdependent the processes described in some publication, the explanation given for its control all too often focusses on regulation by a single or small set of genes, or equivalently signaling pathway signaling pathway proteins and receptors.

The question remains how best to quantitatively and conceptually describe the complex, time-dependent interplay of metabolic, genetic, signaling, and epigenetic factors in adaptation. We hope that by encouraging researchers, and educators, to drop the view that adaptation is controlled at a single level, whether it be genomic, epigenetic, signaling or metabolism, we will expedite achievement of this goal.

## Data Availability

The original contributions presented in the study are included in the article/Supplementary Material, further inquiries can be directed to the corresponding author.

## Abbreviations

ATP, adenosine triphosphate; ADP, adenosine diphosphate; cAMP, cyclic adenosine monophosphate; F1,6P2, fructose 1,6—bisphosphate; G6P, glucose 6-phosphate; Gpr1, G protein receptor 1; HK1, hexokinase 1; HK2, hexokinase 2; PFK1, phosphofructokinase1; PyK2, pyruvate kinase 2F 1; 6P2ase, fructose 1,6—bisphosphatase; PPP, pentose phosphate pathway; PKA, glucose activated cyclic; AMP, protein kinase A pathway; RAS, rat sarcoma signaling pathway.
