# Supplementary material for: The impact of metabolism on the adaptation of organisms to environmental change
Source: Front Cell Dev Biol. 2023 Jun 12;11:1197226. doi: 10.3389/fcell.2023.1197226 (PMC10291235; doi:10.3389/fcell.2023.1197226)
Supplement: Supplementary file 1 [file DataSheet1.PDF]

Supplementary Information

Figure S1. The effect of the glycogen shunt on the concentration control coefficients for G6P and FBP during the first stage of the transition phase and the effect of gene expression on the concentration control coefficients for G6P and FBP at steady state in the Crabtree effect

The figure below, shows the concentration control coefficients for G6P (CG6P) and Fructose 1,6 bisphosphate (CFBP) calculated using Supply and Demand MCA analysis (Hofmeyr and Rohwer, 2011) for yeast undergoing the Crabtree effect. The concentration control coefficient in this case is defined as the proportionality constant between a change in the combined activity of the glycolytic pathway enzymes above FBP and the glycogen shunt (when present).

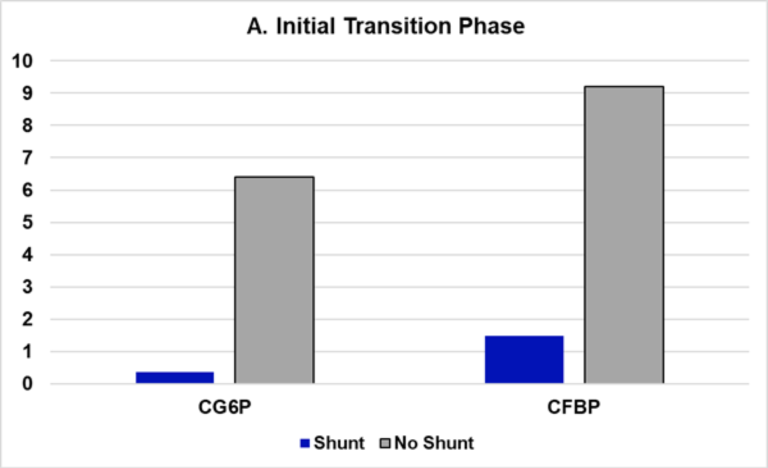

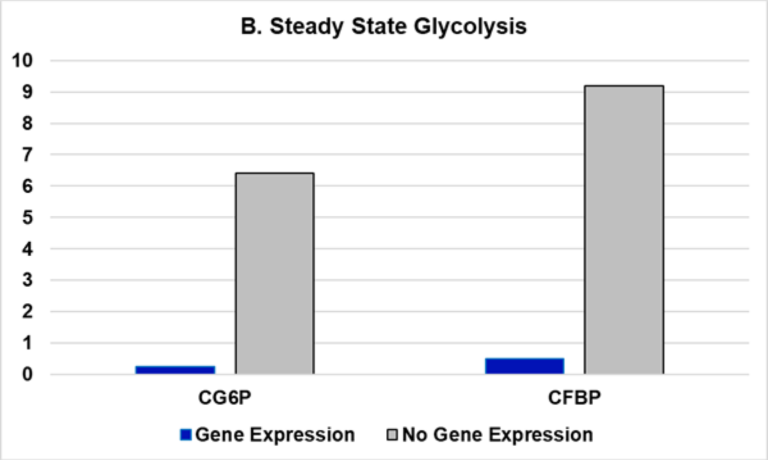

Figure S1A shows the calculated concentration control coefficients respectively with (blue) and without (gray) the Glycogen Shunt during the initial Transition Phase. In the absence of the shunt, a fractional increase in glycolytic enzyme activity, such as hexokinase when glucose concentration is increased, leads to almost a 10 fold higher fractional increase in G6P and FBP than when the shunt is active.

Figure S1B shows the same calculations for the steady state period when the shunt is suppressed. The blue bars are the concentration control coefficients when the new glycolytic enzyme isoforms are expressed and the gray bars when the original isoforms are maintained. Due to the greater sensitivity of the new isoforms to allosteric activation and inhibition the concentration control coefficients are on the order of 10 fold lower. The ability to maintain homeostasis of shared intermediates due to the new isoforms allows the shunt (and futile cycling) to be suppressed, increasing net ATP production per glucose from 0 to the canonical 2 ATPs per glucose.
